# A Brief Overview of Adaptive Designs for Phase I Cancer Trials

**DOI:** 10.3390/cancers14061566

**Published:** 2022-03-18

**Authors:** Anshul Saxena, Muni Rubens, Venkataraghavan Ramamoorthy, Zhenwei Zhang, Md Ashfaq Ahmed, Peter McGranaghan, Sankalp Das, Emir Veledar

**Affiliations:** 1 Center for Advanced Analytics, Baptist Health South Florida, Miami, FL 33176, USA; drvenky37@gmail.com (V.R.); Zhenwei.Zhang@baptisthealth.net (Z.Z.); MdAshfaq.Ahmed@baptisthealth.net (M.A.A.); EmirV@baptisthealth.net (E.V.); 2 Robert Stempel College of Public Health & Social Work, Florida International University, Miami, FL 33199, USA; 3 Miami Cancer Institute, Baptist Health South Florida, Miami, FL 33176, USA; mrube001@fiu.edu; 4 Department of Internal Medicine and Cardiology, Charité—Universitätsmedizin Berlin, Corporate Member of Freie Universität Berlin and Humboldt Universität zu Berlin, 10117 Berlin, Germany; 5 Wellness and Employee Health, Baptist Health South Florida, Miami, FL 33176, USA; SankalpD@baptisthealth.net

**Keywords:** phase I trial, cancer clinical trial, adaptive design, maximum tolerated dose, dose-limiting toxicity, nonparametric designs, algorithm-based designs, parametric model-based designs

## Abstract

**Simple Summary:**

Phase I cancer trials are important for new drug developments to test the safety and optimal dosage of cancer drugs which are usually toxic. Understanding biostatistical methodologies of these designs is important for developing phase I studies that are both safe for the participants and which use optimal dosages for better outcomes. Currently there are several phase I designs that are being refined and modified for better outcomes and newer designs are being continuously developed. In this review article, we described several important phase I study designs to provide a brief overview of existing methods. Our review could be helpful to the research community who intent to have a better and yet a concise summary of existing methods.

**Abstract:**

Phase I studies are used to estimate the dose-toxicity profile of the drugs and to select appropriate doses for successive studies. However, literature on statistical methods used for phase I studies are extensive. The objective of this review is to provide a concise summary of existing and emerging techniques for selecting dosages that are appropriate for phase I cancer trials. Many advanced statistical studies have proposed novel and robust methods for adaptive designs that have shown significant advantages over conventional dose finding methods. An increasing number of phase I cancer trials use adaptive designs, particularly during the early phases of the study. In this review, we described nonparametric and algorithm-based designs such as traditional 3 + 3, accelerated titration, Bayesian algorithm-based design, up-and-down design, and isotonic design. In addition, we also described parametric model-based designs such as continual reassessment method, escalation with overdose control, and Bayesian decision theoretic and optimal design. Ongoing studies have been continuously focusing on improving and refining the existing models as well as developing newer methods. This study would help readers to assimilate core concepts and compare different phase I statistical methods under one banner. Nevertheless, other evolving methods require future reviews.

## 1. Introduction

The phase I clinical trials, also known as first-in-human studies, evaluate the pharmacokinetic and pharmacodynamic properties of clinical drugs as well as their safety and tolerability. Phase I clinical trials are usually done among healthy volunteers who do not have the disease and do not necessarily benefit from the tested drugs. This creates significant dilemma for testing cancer medications because they are highly toxic and could cause significant harm to healthy volunteers. To overcome this ethical problem, phase I trials for cancer medications are usually conducted among cancer patients who have not responded to the standard treatment options. However, this also creates some restrictions as such patients are rare, and studies are small, non-comparative, and single-armed. In addition, such studies are open-labeled and sequential. The primary objective of phase I clinical trials is to calculate and estimate a recommended dose that will be used in efficacy testing during phase II trials. A phase I clinical trial includes many parameters such as starting dose, dose increment, dose decrement, dose-limiting toxicity (DLT), dose-toxicity curve, target toxicity level, dose-efficacy curve, maximum tolerated dose (MTD), optimal biological dose (OBD), and recommended phase II dose (RP2D) [1]. MTD is defined as the highest dose of any drug that will achieve the treatment effect without causing undesirable side effects. DLT is defined as severe and irreversible toxicity caused by the administered drug, preventing further dose escalation. Many advanced statistical studies have proposed novel and robust methods for adaptive designs that have shown significant advantages over conventional dose-finding methods. Artificial intelligence and deep learning methods are also promising for clinical trials, but their applications for phase I trials have not been explored yet [2,3,4,5]. Nevertheless, due to these advanced statistical methods, the number of patients requiring highly toxic or non-efficacious doses has decreased significantly, while statistical efficiency has substantially improved. A relatively small number of concise summaries are available for these advanced statistical methods, but most of the literature is elaborate and extensive. A summary like this would facilitate easier assimilation of concepts and allow a better comparison of different statistical methods all in one place. Therefore, the objective of this study was to provide a summary of such advanced adaptive design methods for phase I cancer trials.

## 2. Nonparametric and Algorithm-Based Designs to Calculate MTD

### 2.1. Traditional *3 + 3* Design

The traditional 3+3 design includes 2 cohorts of 3 patients who are treated with the experimental drug [6,7]. The first cohort receives the dosage of the drug considered safe by animal toxicology studies. Subsequently, the second cohort receives incremental doses calculated using a dose escalation method that follows a modified Fibonacci sequence. In a modified Fibonacci sequence, succeeding dose increment gets smaller with every successive dose increment. This process continues until dose escalations result in at least 2 out of 6 patients starting to experience DLTs. The dose of an experimental drug just below this level is conventionally used as the RP2D. Traditional 3+3 design follows a rule-based design and is based on the assumption that toxicity increases with dose increments. Advantages of this design include the simplicity and easy understandability of the algorithm. In addition, assumptions related to dose-response are not required and the accrual of 3 patients per dose gives further information about the pharmacokinetic inter-patient variability. Nevertheless, the major disadvantage of this design is that it is inflexible, and escalation and de-escalation decisions are based on outcomes from recently recruited patients [8,9]. Other variations in 3+3 design include “3+3+3”, “2+4”, and “3+1+1” (also known as “best of five”) which follow similar methods [10]. In “3+1+1” design an additional patient is added to account for DLTs observed in first cohort of 3 patients and is generally considered a more aggressive method [10].

**Figure 1:**
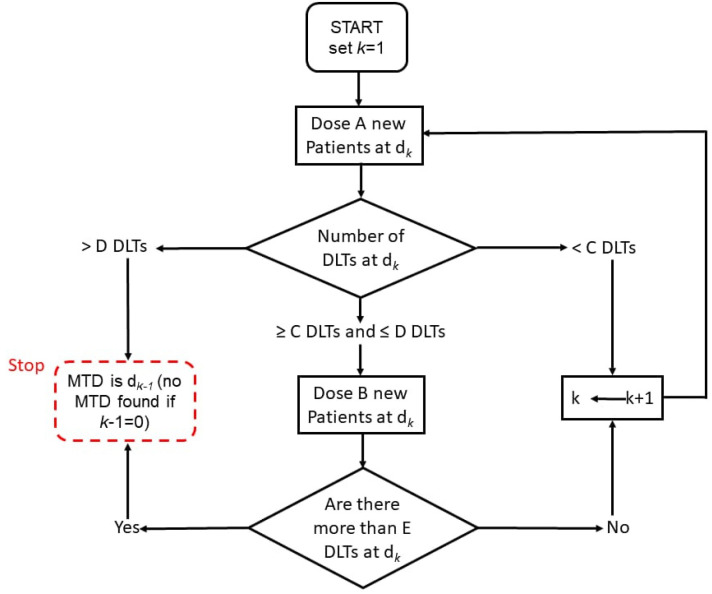
Schematic representation of A+B design without dose de-escalation.

**Figure 2:**
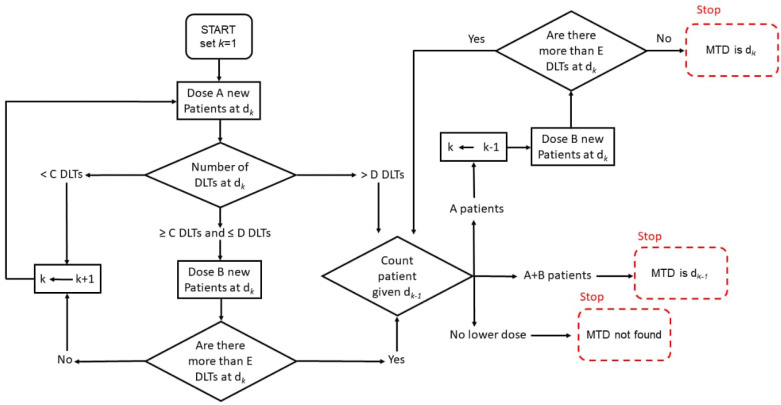
Schematic representation of A+B design with dose de-escalation.

The A+B design got its name from *A* and *B*, which are the numbers of patients for each given dose level [11]. There are 6 parameters used for the complete specification and include A,B,C,D, and *E* as well as an indicator specifying whether the dose escalation or de-escalation is permissible. Parameters *A* to *E* are whole numbers. *A* specifies the total number of patients in the first group that are allocated a dose. *B* specifies the total number of patients in the second group, if needed, who are allocated a dose. *C* specifies the minimum required DLTs in group *A* in order to allocate *B* more. *D* specifies the maximum required DLTs in group *A* in order to allocate *B* more, else the study is stopped or de-escalated. *E* specifies the maximum required DLTs in the A+B group to allow for dose escalation for the next group of *A* patients (Figure 1 and Figure 2). Dose escalation is recommended for the next group when the number of recorded DLTs for a specific dose level is smaller than the lower limit. The study is terminated or the dose de-escalation is recommended for the next group when the number of recorded DLTs for a specific dose level is greater than the upper limit. If the number of recorded DLTs lie between the upper and lower limits, the same dose is recommended for the next group [11]. To develop the conventional 3+3 design, the parameters A,B,C,D, and *E* can be assigned 3,3,1,1, and 1, with dose de-escalation not being permitted [11].

Lin and Shih (2001) investigated statistical properties of the A+B design and formulated the calculation of a dose being chosen as MTD, the expected number of patients treated at each dose level, and the expected number of toxicities at each dose level [12]. In the 3+3 design without dose de-escalation, the probability of MTD is calculated by Equation (1).

(1) $$P\left(MTD=Dose\;i\right)=\left(\prod_{k=1}^{i}\left(P_{0}^{k}+P_{1}^{k}P_{0}^{k}\right)\right)\left(1-P_{0}^{i+1}-P_{1}^{i+1}P_{0}^{i+1}\right),\;1\leq i<n$$

In Equation (1), $P_{0}^{k}=Prob\left(0\mathrm{out}\mathrm{of}3\mathrm{when}\mathrm{treated}\mathrm{at}\mathrm{dose}k\right)={\left(1-p_{k}\right)}^{3}$, and $P_{1}^{k}=Prob\left(1\mathrm{out}\mathrm{of}3\mathrm{when}\mathrm{treated}\mathrm{at}\mathrm{dose}k\right)=3p_{k}{\left(1-p_{k}\right)}^{2}$, and $p_{k}$ is the probability of having DLT at dose level *k*.

### 2.2. Accelerated Titration Designs

To improve the 3 + 3 design, Simon et al. proposed accelerated titration designs (ATDs) based on inferences from 20 phase I clinical trials testing 9 different drugs [13]. ATDs consist of 3 steps: (1) rapid acceleration phase, (2) intra-patient dose escalation phase and (3) assessment of dose-toxicity relationship. In the rapid acceleration phase, patients are treated with different doses of the drug and increments are achieved through single-or double-dose escalations. These increments are stopped either when one occurrence of DLTs or two occurrences of grade 2 toxicities are detected. At this point, traditional 3+3 design is initiated. In the second phase, patients who did not experience toxicities in the first phase receive escalated doses, thus facilitating dose titration. After this phase nonlinear mixed-effects models are used for data analysis and assessment of dose-toxicity relationship which constitutes the third step [13].

The advantages of ATDs are that they decrease the number of patients receiving subtherapeutic doses while at the same time do not significantly increase the number of patients who start showing DLTs. In addition, ATDs decrease the sample size requirements, compared to traditional designs, and in some instances also decrease the duration of the trial. ATDs are also helpful in obtaining more information about the inter-patient variability, dose-toxicity curve, and cumulative toxicity because it relies on dose-toxicity models [13,14].

Assuming that a latent continuous variable is associated with toxicity, data from ATDs allow for model-based analysis [15]. The model, designed to represent different levels of worst toxicity, incorporates parameters for both intrapatient and interpatient variability, and for cumulative toxicity. Let $d_{ij}$ be the dose that *i*th patient receives during dose *j* and receives a total dose $D_{ij}$ for courses prior to *j*. Denoted by α is the effect of cumulative toxicity (α=0 indicates no effect of cumulative toxicity). The random variable $\beta_{i}$ represents interpatient variability in the toxic effects, $\beta_{i}∼N\left(0,\sigma_{\beta }^{2}\right)$. The random variable $\varepsilon_{ij}$ represents intrapatient variability in the toxic response, $\varepsilon_{ij}∼N\left(0,\sigma_{\epsilon }^{2}\right)$. Then the latent magnitude $y_{ij}$ of the worst toxicity for patient *i* in course *j* be formulated through Equation (2)

(2) $$y_{ij}=log\left(d_{ij}+\alpha D_{ij}\right)+\beta_{i}+\epsilon_{ij}$$

Corresponding to the toxicity grading, with assumed cut-off parameters, following are the different grades of toxicities

(3) $$\begin{array}{cc} y_{ij}\leq K_{1}\; & :\mathrm{grade}0-1\mathrm{toxicity} \end{array}$$

(4) $$\begin{array}{cc} K_{1}<y_{ij}\leq K_{2} & :\mathrm{grade}2\mathrm{toxicity} \end{array}$$

(5) $$\begin{array}{cc} k_{2}<y_{ij}\leq K_{3} & :\mathrm{grade}3\mathrm{toxicity} \end{array}$$

(6) $$\begin{array}{cc} y_{ij}>K_{3}\; & :\mathrm{grade}4\mathrm{toxicity} \end{array}$$

This model is a generalization of the $K_{max}$ model of Sheiner, Beal, and Sambol [16].

### 2.3. Bayesian Algorithm-Based Designs

Ji, Li, and Bekele developed a method for finding toxicity data using Bayesian decision rules based on toxicity posterior intervals (TPI) [17]. Some of the advantages of this method include its simplicity of implementation, transparency, and statistical robustness. In this design, a beta-binomial model is used for estimating toxicity probabilities at each dose level. For evaluating the probability of toxicity at each dose level, this design assumes a beta-binomial model. It is based on TPI. At any dose level, for a given target toxicity and toxicity outcomes, the posterior probabilities (PP) of three non-overlapping intervals corresponding to dose escalation, dose de-escalation, and the initial doses are evaluated. The decision for the next cohort is made after determining the interval with the highest PP. The algorithm stops as soon as the MTD is exceeded. Moreover, it also has an exclusion rule prohibiting escalation to doses with higher toxicity probabilities. The isotonic-transformed posterior toxicity probability, which is closest to the target is assigned as MTD [17].

A modified TPI method based on the unit probability mass statistic was proposed by Ji and colleagues [18]. This modified design has advantages over the traditional 3+3 design. Specifically, the mean number of patients treated above MTD is lower and the evaluated MTD is more accurate. The modified TPI includes many prominent features of TPI model as well as its advantages. In addition, modified TPI is simpler because it only necessitates equivalence interval condition, where any dose with toxicity probability within the equivalence interval can be assumed as MTD.

The target toxicity probability is denoted by $p_{T}$. Considering an equivalence interval $\left[p_{T}-\epsilon_{1},p_{T}+\epsilon_{2}\right]$ with $\epsilon_{1}$ and $\epsilon_{2}$ being elicited from expert physicians, the entire range of possible values for the probability of toxicity at dose *i*, $p_{i}$, can be broken down into the following intervals:

- The underdosing interval (UI), defined as $\left(0,p_{T}-\epsilon_{1}\right)$
- The equivalence interval (EI), defined as $\left(p_{T}-\epsilon_{1},p_{T}+\epsilon_{2}\right)$
- The overdosing interval (OI), defined as $\left(p_{T}+\epsilon_{2},1\right)$

These intervals are mutually exclusive and mutually exhaustive.

For a continuous random variable *X* with cumulative probability mass function F(x) (i.e. Pr(X<x)=F(x)), the unit probability mass (UPM) is defined for an interval (a,b) to be (F(b)−F(a))/(b−a). The UPM is the probability mass in an interval divided by the width of the interval and can be interpreted as the average probability density of the interval. Using the posterior distribution identified above, we can calculate the three UPMs using Equations (7)–(9)

(7) $$\begin{array}{cc} UPM_{UI} & =Pr\left(p_{i}\in UI\right)/\left(p_{T}-\epsilon_{1}\right), \end{array}$$

(8) $$\begin{array}{cc} UPM_{EI} & =Pr\left(p_{i}\in EI\right)/\left(\epsilon_{1}+\epsilon_{2}\right), \end{array}$$

(9) $$\begin{array}{cc} UPM_{OI} & =Pr\left(p_{i}\in OI\right)/\left(1-p_{T}+\epsilon_{2}\right). \end{array}$$

The logical action in the dose-finding trial depends upon which of the above three UPMs is the greatest. If $UPM_{UI}>UPM_{EI}$, $UPM_{OI}$, then the current dose is likely an underdose, and it should be escalated to dose to i+1. In contrast, if $UPM_{OI}>UPM_{UI}$, $UPM_{EI}$, then the current dose is likely an overdose and it should be de-escalated to dose to i−1 for the next patient. If $UPM_{EI}>UPM_{UI}$, $UPM_{OI}$, then the current dose is deemed sufficiently close to $p_{T}$ and it is preferable to stay at dose-level *i*.

Furthermore, the following rule should be used to avoid recommending dangerous doses. A dose is deemed inadmissible for being excessively toxic if for a certainty threshold, ξ.

(10) $$Pr(p_{i}>p_{T}|data)>\xi$$

If a dose is excluded by this rule, it should not be recommended by the model. Irrespective of the values of $UPM_{UI}$, $UPM_{EI}$ and $UPM_{OI}$, the design will recommend staying at dose *i*, rather than escalating to a dose previously identified as being inadmissible. Furthermore, the design will advocate stopping when the lowest dose is inferred to be inadmissible. In estimation, independent uniform prior for $p_{i}$ can be assumed, while assuming toxicity increases with dose levels, i.e., $p_{1}<p_{2}<...<p_{d}$ for increasing dose level i=1,2,…,d.

### 2.4. Up-and-Down Designs

A number of up-and-down designs such as biased coin, improved up-and-down, group up-and-down, cumulative group up-and-down, and up-and-down designs with delayed response have been recommended for improving the operating features of the traditional 3+3 design [19]. However, there methods are rarely used in practice.

The biased coin design is more flexible than traditional 3+3 design because it can accommodate any specified toxicity rate based on random walk theory [20,21]. However, the biased coin design is limited due to its lower efficiency because it relies on the outcomes of the most recently treated patients and disregards the information from previously treated patients. Biased coin design assigns patients one at a time to a specified dose level, whereas it is easier to treat patients as cohort for practical purposes. In order to overcome this disadvantage, the group up-and-down designs were developed. Group up-and-down designs are non-randomized methods, where dose modifications (escalations, de-escalations) are estimated using the toxicity values of the most recently tested cohorts [22]. More specifically, dose modifications are conditioned by the cohort size. The toxicity numbers within a cohort are chosen in such a way that a desired target toxicity probability could be achieved. Group up-and-down designs use additional factors such as sample size and cutoff points for the number of toxicities in the cohort, enabling us to estimate additional toxicity probability.

Up-and-down designs with delayed responses were developed for phase I trials with lengthy assessment periods, where succeeding patients are enrolled into the study before toxicity results from preceding patients become available. In this design, successive patients are assigned the dose estimated from completely evaluated patients and not necessarily from the previously enrolled patients [23]. Advantages include decreased study duration and cost reduction while maintaining agreeable levels of statistical power. This model was further adapted to account for patients’ follow-up time for decreasing delays in dose assessments. Hence, this design is also known as “adaptive accelerated biased coin design” [24].

The cumulative group up-and-down design was developed to summarize toxicity information from cumulative number of patients treated by a dose before decisions are made for escalations or de-escalations [25]. For estimating doses for new cohorts, this design uses the same principles as group up-and-down design but considers the cumulative number of patients receiving the dose as cohort size. The cumulative group up-and-down design has been considered as the best up-and-down design because of its safety, high MTD selection percentage, and greater number of patients assigned to the MTD [19,26]. The cumulative group up-and-down design performs almost similar to optimal group up-and-down design. Though performance improvements are generally not possible with cumulative group up-and-down designs, some specific instances such as steeply increasing dose-toxicity curves, or plateauing curves followed by steeply increasing curves are examples where this design could be used for further improvements. However, in these instances, the cumulative group up-and-down design selects doses much below the MTDs compared to other nonparametric designs. In order to overcome this shortcoming, asymmetric lower and higher cutoffs are used for achieving greater levels of dose escalations [19]. In addition, distributing patient data across the doses increases the chances of identifying the MTDs from its surrounding doses. Though up-and-down designs have been recommended by many researchers for their simplicity and robustness, currently they are not popular. Future Phase I trials should consider using these designs for improving outcomes.

Let $F(d_{i})$ be the true toxicity rate at dose $d_{i}$. The transition rules for each up-and-down design can be converted to transition probabilities, given the knowledge of *F* at all the doses. Subsequently, theoretical properties of Markov process on discrete state space can be applied. The key property underlying up-and-down designs’ usefulness is that the escalation (‘up’) probabilities decrease with increasing dose, while the de-escalation (‘down’) probabilities increase with decreasing dose. This property is a direct consequence of the monotone increasing nature of *F*. The sequence of doses assigned to the experiment’s *n* patients, $x_{1},...,x_{n}$, is a Markov chain with a central tendency. Asymptotically, the sequence will revolve around a central tendency. Oron and Hoff called this point as the balance point $d^{*}$, the quantile at which the two probabilities are equal [27]. The toxicity rate at $d^{*}$ will be denoted as $p^{*}$. It might be identical to the target rate $p_{T}$, and $p_{T}$ is set according to the experimental goals, whereas $p^{*}$ is a property of the chosen design. It suffices that $p^{*}\approx p_{T}$. For a group up-and-down design, $p^{*}$ is the solution of equation (11) with *C* and *D* as defined above.

(11) $$\mathrm{Pr}\left\{Binomial\left(s,p^{*}\right)\leq C\right\}=\mathrm{Pr}\left\{Binomial\left(s,p^{*}\right)\geq D\right\}$$

Except for special cases, the solution has to be found numerically via a simple root-finding algorithm. Asymptotically, dose assignment will be strongly clustered on doses close to the balance point [20].

### 2.5. Isotonic Designs

Leung et al. in 2001 proposed isotonic designs based on isotonic regression methods [28]. The isotonic designs are based on the assumption that the toxicities do not decrease with doses. These designs use isotonic regressions for the collected data. Throughout the trial, patients are administered the doses that are considered close to the MTDs. Isotonic designs doubly improve standard designs through summarizing the risks for toxicities for given doses and through isotonic regressions for characterizing dose-toxicity associations. The advantages of isotonic designs include that they can estimate the MTDs with differential target toxicity levels, they are robust and easy to implement, they constitute a semiparametric method, they are based on the assumption that doses and toxicities are monotonically related, and they are ideal for groups of collective drugs and treatments [29]. However, the accuracy of MTDs estimated by isotonic designs are much lower than other model-based designs such as continual reassessment method (CRM) and escalation with overdose control (EWOC; described below) designs. Many studies have applied modifications to isotonic designs for phase I trials [30,31,32]. For example, pool-adjacent-violators algorithm (PAVA) are used in addition to isotonic regression methods to estimate the probabilities of MTDs for each dose level [30]. Chen et al. used quasi-continuous toxicity scores for estimating MTDs in situations where parametric dose-toxicity relationships are not adequately clear [33]. However, Ivanova et al. has cautioned that isotonic designs using one-parameter models overestimates the doses, and hence the parameter should be selected cautiously using Markov chain theory which ensures better assignment of the MTD and nearby doses [34].

Numerically, for any dose $d_{r}$ below $d_{i}$ (r≤i) and any dose $d_{s}$ above $d_{i}$ (s≥i), the pooled estimate of risk can be assessed by Equation (12)

(12) $$w_{r,i,s}=\frac{\sum_{j=r}^{s}\mathrm{number}\mathrm{of}\mathrm{toxicities}\mathrm{at}d_{j}}{\sum_{j=r}^{s}\mathrm{number}\mathrm{of}\mathrm{tested}\mathrm{at}d_{j}},\;1\leq r,i,s\leq k$$

The risk $q_{i}$ at dose $d_{i}$ can be estimated using the isotonic regression Equation (13)

(13) $${\hat{q}}_{i}=\min_{\left[i\leq s\leq k\right]}\;\max_{\left[l\leq r\leq i\right]}w_{r,i,s}$$

The ${\hat{q}}_{i}$ must be at least as large as any of $w_{1,i,s},w_{2,i,s},...,w_{i,i,s}$ (or the maximum of these) for any *s*(s≥i). Similarly, ${\hat{q}}_{i}$ must be smaller than any of $w_{r,i,i},w_{r,i,i+1},...,w_{r,i,k}$ (or the minimum of these) for any *r*(r≤i). Decision rules for escalating or de-escalating dose for the next cohort in the isotonic design may be different. Ivanova and Flournoy have extensively described the comparison among the different decision rules [35].

## 3. Parametric Model-Based Designs to Calculate MTD

### 3.1. Continual Reassessment Method (CRM)

The Continual Reassessment Method (CRM) constitutes one of the earliest Bayesian model-based designs for phase I trials that allow for multiple dose escalations and de-escalations [36]. In this method, the initial dose-efficacy curve characteristics are estimated from animal studies or clinical studies. All patients are administered doses that are closest to the MTD, and the probabilities of experiencing dose-limiting toxicity are calculated continuously, until a prespecified condition is reached. Upon reaching this condition, the trial is stopped. Though there are many stopping rules, the common ones include stopping the trial when 6 patients are given the same dose, or when pre-estimated probabilities of dose-limiting toxicity are achieved [36]. This method has not received wider acceptance because it could accidently expose patients to toxic doses when the prespecified conditions are inaccurately estimated. To overcome this shortcoming, many modifications have been proposed to the original CRM. For example, in one such method, the first patient is administered the lowest calculated starting dose estimated from the results of animal studies [37]. In addition, methods such as dose escalations with prespecified levels, preventing dose escalations for consecutive patients when preceding patient experience dose-limiting toxicities, maintaining the same doses without escalations for many patients when high dose levels are reached, and increasing the number of patients in the cohort who are being treated at R2PD levels, are also generally followed [38]. These modifications have significantly improved the use of CRM for phase I clinical trials.

Let F(d,β) be the dose toxicity model that is strictly increasing in the dose *d* for all β. Common choices for the dose toxicity model are estimated through Equations (14) and (15).

- Empiric:
  (14) $$F\left(d,\beta \right)=d^{\beta }$$
- One-parameter logistic:
  (15) $$F\left(d,\beta \right)={\left\{1+\exp\left(-\alpha +\beta d\right)\right\}}^{-1}$$

In Equation (15), α is a fixed constant.

In the Bayesian framework, a prior distribution on the model parameter β is assumed. Given the prior distribution and the data accrued up to the first *n* patients (i.e., the doses assigned to the patients and their corresponding toxicity outcomes), β can be estimated by the posterior mean (denoted as ${\hat{\beta }}_{n}$). The dose level recommended for the (n+1) th patient is the dose with the model based DLT probability closest to $p_{T}$ as estimated through Equation (16)

(16) $$\arg\min_{d_{i}}\left|F\left(d_{i},{\hat{\beta }}_{n}\right)-p_{T}\right|$$

This process continues until a pre-specified number of subjects are accrued.

### 3.2. Escalation with Overdose Control

Escalation with overdose control (EWOC) method was proposed and developed by Babb, Rogatko, and Zacks, to overcome the limitations of continual reassessment method such as higher number of patients being exposed to toxic doses [39]. This method uses a Bayesian adaptive dose finding design for estimating dose escalations for successive patients while decreasing the probability of patients exposed to toxic doses. One of the salient characteristics of this model is that the number of patients receiving doses above MTDs are already estimated using a feasibility bound recommendation by physicians with cautions for toxic doses [40]. Subsequently, in the course of the phase I trial, the probabilities of doses that exceed the MTD are estimated for each patient and dose escalations are prohibited if these probabilities are greater than prespecified values. In EWOC method, the succession of dose escalations starts approaching probabilities of MTDs. This results in all enrolled patients beyond a specific time receiving doses very close to the MTDs [41]. EWOC significantly improves the accuracies of MTDs and hence the efficiency of phase I trials.

Let MTD, γ, be the dose expected to produce some degree of DLT in a specified proportion θ of patients as shown in Equation (17)

(17) Pr{DLT|dose=γ}=θ,

The dose-toxicity relationship is modeled parametrically through Equation (18)

(18) $$P\left(Y=1|\mathrm{dose}=x\right)=F\left(\beta_{0}+\beta_{1}x\right)$$

In the model, $\beta_{1}>0$ so that the probability of a DLT is a monotonic increasing function of doses. Assuming a logistic distribution for *F* and $\rho_{0}$ being the starting dose, the dose-toxicity relationship is modeled through Equation (19)

(19) $$P\left(Y=1|dose=x\right)=\frac{\exp\left\{\ln\left(\frac{\rho_{0}}{1-\rho_{0}}\right)+\ln\left(\frac{\theta (1-\rho_{0})}{\rho_{0}\left(1-\theta \right)}\right)\frac{x}{\gamma }\right\}}{1+\exp\left\{\ln\left(\frac{\rho_{0}}{1-\rho_{0}}\right)+\ln\left(\frac{\theta (1-\rho_{0})}{\rho_{0}\left(1-\theta \right)}\right)\frac{x}{\gamma }\right\}}$$

Suppose subject *i* was administered dose $x_{i}$ and observed response $y_{i}$, which would be 1 for exhibiting DLT and 0 otherwise, then Equation (20) can be used to estimate the dose after observing *k* patients.

(20) $$D_{k}=\left\{\left(x_{i},y_{i}\right),i=1,...,k\right\}$$

Let $\prod_{k}x$ be the marginal posterior distribution of γ, given $D_{k}$, then, at a feasibility bound α for the posterior probability of exceeding the MTD, the dose received by *k*th patient can be estimated using the Equation (21)

(21) $$x_{k}=\prod_{k}^{-1}\left(\alpha \right)$$

The corresponding sequence of doses generated by this design extends up to the unknown MTD while also minimizing the amount by which patients are underdosed. Calculation of the marginal posterior distribution of γ is performed using numerical integration.

### 3.3. Bayesian Decision Theoretic and Optimal Designs

In addition to the above designs, there are Bayesian decision theoretic and optimal designs for phase I cancer trials which include formal optimality criteria while designing adaptation rules [42]. Some of these designs include Bayesian decision theoretic designs, Bayesian optimal sequential designs, and Hybrid designs. The Bayesian decision theoretic framework developed by Whitehead and Brunier is based on loss/gain functions, a collection of possible doses assigned for patients, prior distribution of model parameters, and recommendation of a specific dose-toxicity model [43]. Haines et al. developed the Bayesian optimal sequential designs using optimal design theory for phase I trials based on the Bayesian c- and D-optimality criteria [44]. An important aspect of this deign includes the creation and application of a constraint that limits the doses that will be dispensed to the patients. This constraint will be based on a maximum acceptable dose which includes the support points and weights of the candidate design [44]. In order to overcome the “treatment versus experimentation” dilemma while designing phase I trials, Bartroff and Lai proposed the hybrid design [45]. This design involves a dynamic programing which may overcome the limitations of what is acceptable through computation alone. They proposed a hybrid design that combines treatment designs like EWOC and learning designs like D-optimal design. In this design, the adaptive weight is initially skewed towards learning designs and eventually starts to skew towards treatment designs as the study progresses [45]. In spite of the advantages of these designs the current review could not describe them in detail because it is beyond the scope of this review.

## 4. Conclusions

In the current review, we present a brief, though not exhaustive list of novel techniques for finding dosages that are appropriate for phase I cancer trials. Adaptive designs are increasingly being used in phase I cancer trials, particularly during the early stages of clinical trials. The purpose of phase I studies is to estimate the dose-toxicity profile of the drugs and to select suitable doses for subsequent studies. It is different from confirmatory trials which are primarily driven by hypothesis testing. Since there is limited information about the drug characteristics at the beginning of clinical drug development, adaptive research designs are appropriate for estimating the doses in phase I trials. Although adaptive designs for phase I cancer trials have made significant advances in the last decade, the traditional 3+3 design is still the most popular design. Nevertheless, there are continued developments in refining the existing models as well as designing newer methods for better estimation of drug doses in phase I cancer trials. Therefore, future reviews should explore these options.

## Data Availability

Not applicable.
